# Effect of resistance to third-generation cephalosporins on morbidity and mortality from bloodstream infections in Blantyre, Malawi: a prospective cohort study

**DOI:** 10.1016/S2666-5247(22)00282-8

**Published:** 2022-12

**Authors:** Rebecca Lester, Patrick Musicha, Kondwani Kawaza, Josephine Langton, James Mango, Helen Mangochi, Winnie Bakali, Oliver Pearse, Jane Mallewa, Brigitte Denis, Sithembile Bilima, Stephen B Gordon, David G Lalloo, Christopher P Jewell, Nicholas A Feasey

**Affiliations:** a Malawi-Liverpool Wellcome Research Programme, Kamuzu University of Health Sciences, Blantyre, Malawi; b Department of Paediatrics and Child Health, Kamuzu University of Health Sciences, Blantyre, Malawi; c Department of Medicine, Kamuzu University of Health Sciences, Blantyre, Malawi; d Department of Clinical Sciences, Liverpool School of Tropical Medicine, Liverpool, UK; e Parasites and Microbes Programme, Wellcome Sanger Institute, Hinxton, UK; f Department of Medicine, Queen Elizabeth Central Hospital, Blantyre, Malawi; g Centre for Health Informatics, Computing and Statistics, Lancaster University, Lancaster, UK

## Abstract

**Background:**

The burden of antimicrobial resistance is a major threat to global health; however, prospective clinical outcome data from Africa are scarce. In Malawi, third-generation cephalosporins are the antibiotics of choice in patients admitted to hospital despite a rapid proliferation of resistance to these drugs. We aimed to quantify the effect of resistance to third-generation cephalosporins on mortality and length of hospital stay among patients with bloodstream infections.

**Methods:**

We did a prospective cohort study of patients admitted to Queen Elizabeth Central Hospital in Blantyre, Malawi. Patients of all ages who had positive blood cultures for Enterobacterales were included, with the exception of those from the genus *Salmonella*, and were followed up for 180 days. We characterised blood culture isolates using whole-genome sequencing and used Cox regression models to estimate the effect of resistance to third-generation cephalosporins on length of hospital stay, in-hospital mortality, and survival.

**Findings:**

Between Jan 31, 2018, and Jan 13, 2020, we recruited 326 patients, from whom 220 (68%) of 326 isolates were resistant to third-generation cephalosporins. The case fatality proportion was 45% (99 of 220) in patients with bloodstream infections that were resistant to third-generation cephalosporins, and 34% (36 of 106) in patients with bloodstream infections that were sensitive to third-generation cephalosporins. Resistance to third-generation cephalosporins was associated with an increased probability of in-hospital mortality (hazard ratio [HR] 1·44, 95% CI 1·02–2·04), longer hospital stays (1·5 days, 1·0–2·0) and decreased probability of discharge alive (HR 0·31, 0·22–0·45). Whole-genome sequencing showed a high diversity of sequence types of both *Escherichia coli* and *Klebsiella pneumoniae*. Although isolates associated with death were distributed across clades, we identified three *E coli* clades (ST410, ST617, and ST648) that were isolated from 14 patients who all died.

**Interpretation:**

Resistance to third-generation cephalosporins is associated with increased mortality and longer hospital stays in patients with bloodstream infections in Malawi. These data show the urgent need for allocation of resources towards antimicrobial resistance mitigation strategies in Africa.

**Funding:**

Wellcome Trust and Wellcome Asia and Africa Programme.

## Introduction

The burden of antimicrobial resistance is predicted to be high in sub-Saharan Africa, but the sparsity of data linking laboratory results to patient outcomes limits estimates of the burden of disease.^1^, ^2^ Bacteria that are resistant to third-generation cephalosporins have been highlighted by WHO as pathogens of major importance,^3^ particularly in sub-Saharan Africa, where there is widespread reliance on ceftriaxone for the treatment of severe infections.^4^, ^5^

Sentinel surveillance of patients with bacteraemia at Queen Elizabeth Central Hospital (QECH) in Blantyre, Malawi, over the past 25 years has shown a rapid proliferation of resistance to third-generation cephalosporins among Enterobacterales that cause bloodstream infections since the roll-out of ceftriaxone in 2005.^6^ The poor availability and prohibitive cost of alternatives to ceftriaxone means that, unlike in high-income countries, these infections are often untreatable. However, despite the need to improve estimates of the antimicrobial resistance burden and to appropriately prioritise allocation of resources to antimicrobial resistance mitigation strategies, data on the effect of this increase in resistance to third-generation cephalosporins on patients and health systems in Malawi are unavailable.^1^, ^7^

To address these data gaps, we aimed to estimate the morbidity and mortality outcomes of patients with bloodstream infections in Blantyre, Malawi, caused by Enterobacterales that are resistant or sensitive to third-generation cephalosporins. Our specific objectives were to estimate the effect of resistance to third-generation cephalosporins on in-hospital mortality, 6-month survival, and length of hospital stay. Through the use of whole-genome sequencing, we also describe the molecular epidemiology of *Escherichia coli* and *Klebsiella pneumoniae* isolated from patients with bloodstream infections.

**Research in context**

**Evidence before this study**

We previously conducted a systematic review of the prevalence and outcomes of bloodstream infections resistant to third-generation cephalosporins in sub-Saharan Africa, searching for papers published between Jan 1, 1990, and Dec 21, 2017, with no language restrictions. By updating the search to March 1, 2021, we added two further studies. We searched PubMed with the search terms“antimicrobial resistance” AND “drug resistance, bacterial” AND “bloodstream” OR “bacteremia” AND a search string that included all sub-Saharan African countries as defined by the UN list of 54 African sovereign states. We found only two prospective studies designed to identify mortality attributed to resistance to third-generation cephalosporins as a primary outcome. Both studies were in children from Senegal and found that bloodstream infection resistant to third-generation cephalosporins remained the only significant independent risk factor for death. However, a 2021 retrospective multisite analysis from South Africa found no effect of resistance to third-generation cephalosporins on patient outcomes. The most comprehensive analysis of the antimicrobial resistance burden worldwide was a modelling study that used all available data. This modelling study estimated that deaths from antimicrobial resistance will be high in sub-Saharan Africa, but identified important data gaps in low-income settings, and no clinical outcome data were available from Malawi.

**Added value of this study**

We found that outcomes of culture-confirmed Enterobacterales bloodstream infection are unacceptably poor in Malawi, and that resistance to third-generation cephalosporins is associated with increased mortality and longer hospital stays. Using standardised methodology approved by WHO to generate our estimates, we provide the only detailed health outcome data to our knowledge on resistance to one of the most commonly used antibiotics in hospitalised patients in Malawi, and some of the first estimates of the antimicrobial resistance health-care burden in sub-Saharan Africa.

**Implications of all the available evidence**

The prevalence of resistance to third-generation cephalosporins among bloodstream Enterobacterales in sub-Saharan Africa is high, and access to alternative, WHO Watch and Reserve group antibiotics needed to treat these infections is poor. Bloodstream Enterobacterales are associated with very high mortality irrespective of antimicrobial susceptibility, but resistance to third-generation cephalosporins is associated with increased mortality and longer hospital stays. Future research should combine clinical outcomes with population incidence data for Malawi to generate burden estimates for this country. Ultimately, clinically orientated surveillance that links routinely captured laboratory data to patient outcomes is needed, as are pragmatic trials of the management of sepsis.

## Methods

### Study design and participants

This prospective, longitudinal, observational cohort study recruited patients from QECH in Blantyre, Malawi. Malawi is a low-income country with low health-care expenditure (approximately 3% of gross domestic product).^8^ In 2021, the prevalence of HIV in adults was 7·7% and an estimated 58 000 children aged 0–14 years were living with HIV.^9^ QECH is a 1300-bed teaching hospital that provides free inpatient care to Blantyre (estimated population 800  064) and tertiary care to the surrounding districts. The hospital receives approximately 10  000 adult and 30  000 paediatric admissions per year. The number of patients admitted to QECH with HIV infection decreased between 2012 and 2019, but the prevalence of HIV in Blantyre remains high at 53 per 100  000 residents in 2019.^10^

Patients of all ages were eligible for recruitment if their blood culture was positive for bacteria of the order Enterobacterales, with the exception of those from the genus *Salmonella*. Patients were excluded if they were unable to consent and had no representative (guardian) available to provide proxy consent, or if they spoke neither English nor Chichewa. For patients who had died by the time their blood culture results were known, a waiver of consent was in place to enable the inclusion of their anonymised data.^11^ Patients with salmonellae infections were excluded because resistance to third-generation cephalosporins in non-typhoidal *Salmonella* is rare and has not yet been reported in *Salmonella enterica* serotype Typhi in Malawi.

Participants were followed up throughout their stay in hospital until discharge or death, allowing for measurement of in-hospital mortality. To allow for survival analysis and calculation of 6-month mortality, patients or their families were telephoned at 28 days, 3 months, and 6 months after discharge. If a patient had died, family members were asked the date of death.

Ethical approval for the study was granted by the University of Malawi College of Medicine Research Ethics Committee (protocol number P.10/17/2299) and by the Liverpool School of Tropical Medicine Research Ethics Committee (protocol number 17-063). The Liverpool School of Tropical Medicine acted as the study sponsor. Written informed consent was obtained from study participants or their guardian if the patient was unable to consent, and from a parent or guardian if the patient was younger than 18 years.^11^ In reporting this study, we followed the Strengthening the Reporting of Observational Studies in Epidemiology guidelines.^12^ A detailed study protocol has been published.^11^

### Procedures

The Malawi-Liverpool Wellcome Research Programme (MLW) diagnostic microbiology service provides free blood and cerebrospinal fluid culture testing to patients admitted to the adult and paediatric wards at QECH. Blood cultures were processed at the MLW laboratory, which is quality-assured by the UK National External Quality Assessment Service, and were reviewed daily on weekdays to identify cultures from patients that were positive for Enterobacterales (with the exception of *Salmonella* spp); positive cultures from the weekend were identified on a Monday morning. For patients with positive blood cultures, we collected data on the age and sex of the participants; data on the use of health-care services before the current admission to hospital, including previous hospitalisation, surgery, catheterisation, and antibiotic use; and clinical data including antibiotic treatment during the current hospital admission. The medical records of patients who had died were reviewed and data were captured using the same study case report forms as for participants who were alive. Bloodstream infections were categorised according to their suspected location of onset (community or hospital) and their association with health-care facilities (appendix 1 p 1). Patients were followed up for 180 days after bloodstream infection. Data were collected using Open Data Kit software (ODK; version 1.4.10). Completed ODK forms were sent daily to a dedicated secure SQL database.

Isolates were identified using the Analytical Profile Index. The susceptibility of bacteria to the antibiotics available at QECH was established using the disc diffusion method (EUCAST version 7.0).^13^ For Enterobacterales that were resistant to one or both of cefpodoxime or ceftriaxone, extended-spectrum β-lactamase production was confirmed using the species-dependent combination disc method.

### Genomic characterisation of isolates

We used whole-genome sequencing to characterise *E coli* and *K pneumoniae* isolates to investigate the presence of hypervirulent lineages. Genomic DNA was extracted from bacterial isolates at MLW (appendix 1 p 5). The extracted DNA was sequenced at the Wellcome Sanger Institute (Hinxton, UK) on the Illumina X10 platform (Illumina, San Diego, CA, USA) to generate 150 base-paired end reads. Raw sequenced data were deposited in the European Nucleotide Archive (appendices 2 and 3). To characterise the genomes, we conducted in silico multilocus sequence typing and phylogenomic analysis using IQ-TREE. We also screened for the presence and absence of antimicrobial resistance genes using ABRIcate (appendix 1 p 5).

### Outcomes

The primary outcome measure was in-hospital mortality (measured as a proportion of all patients in the cohort), and secondary outcomes were 6-month survival and length of hospital stay (in days).

### Statistical analysis

All analyses were carried out in R, version 4.0.2. Sample size considerations are described in the study protocol.^11^ In brief, the study was powered to detect a difference in the proportions of in-hospital mortality between participants with bloodstream infections that were resistant and those that were sensitive to third-generation cephalosporins. We estimated that a sample size of 250 participants would provide 80% power to detect a difference between 28-day mortality rates of 10·0% and 24·1%. The sample size target was increased to 350 patients to account for attrition.

The primary outcome of in-hospital mortality was calculated as case fatality proportions, with exact binomial 95% CIs, stratified by third-generation cephalosporin resistance status. For the secondary outcome of 6-month survival, Kaplan-Meier estimates of the survival function over the study period were generated, stratified by third-generation cephalosporin resistance status. Day 0 was the day of infection, which was assumed to be the day of blood-culture sampling. Observations were right-censored at the end of the study follow-up period (180 days). The log-rank test was used to test for differences in survival according to third-generation cephalosporin resistance status.

We used Cox regression models to establish the effect of resistance to third-generation cephalosporins on in-hospital mortality. We first hypothesised a causal structure to identify potential confounders in the models, using a directed acyclic graph (appendix 1 p 4) to represent the likely relationships between resistance to third-generation cephalosporins and death. The directed acyclic graph was used to select covariates for logistic regression models, which were both fitted for each set of data. In model 1, death (as the outcome) was regressed onto third-generation cephalosporin resistance status, with health-care exposures and age included as adjusting covariates. Model 2 was the same as model 1, but with the addition of effective antibiotic use as an adjusting covariate.

In model 1, we estimated the total effect of resistance to third-generation cephalosporins on death, and in model 2 we included a covariate for whether the administered antibiotic was effective against the bacterial isolate from bloodstream infection. This approach allowed us to control for the confounding effect of antibiotic susceptibility, and therefore estimate the direct effect of resistance to third-generation cephalosporins on death. The approach to variable selection is further described in appendix 1 (p 4). Model outputs are presented as hazard ratios (HRs) with 95% CIs.

For the secondary outcome regarding length of hospital stay, Kaplan-Meier curves were generated using the day of infection to the day of discharge from hospital as the timeframe, stratified by susceptibility status, to display differences in the length of stay. To identify the association between resistance to third-generation cephalosporins and length of hospital stay, Cox models were constructed using the day of infection to the day of discharge as the timeframe. Two models were constructed as per the mortality models: the outcome variable was length of hospital stay, and the explanatory variables were health-care exposures and age in model 1, and the same but with the addition of effective antibiotic use in model 2. Observations were right-censored if death occurred before discharge from hospital, and HRs for discharge were presented with 95% CIs.

All models were sensitivity-tested for entry point, using the day of hospital admission as the model entry point instead of the day of infection.

### Role of the funding source

The funders of the study had no role in study design, data collection, data analysis, data interpretation, or writing of the report.

## Results

Between Jan 31, 2018, and Jan 13, 2020, we recruited 326 patients (table 1, appendix 1 p 3). Of these, 220 (67%) had bloodstream infections that were resistant to third-generation cephalosporins and 106 (33%) had bloodstream infections that were sensitive to third-generation cephalosporins. Of the 326 participants, 226 (69%) were alive at the time of recruitment and 100 (31%) had died by the time the blood culture result was known. The most common pathogens isolated from blood cultures were *E coli* (identified in 156 [48%] of 326 isolates) and *K pneumoniae* (identified in 130 [40%] of 326 isolates; table 2).

**Table 1:** Baseline characteristics of patients

		**Third-generation-cephalosporin resistant**	**Third-generation-cephalosporin sensitive**	**Overall**
Age, years		3 (1 month–37 years)	32 (4–55)	13 (1–42)
Sex				
	Female	107/220 (49%)	51/106 (48%)	158/326 (48%)
	Male	113/220 (51%)	55/106 (52%)	168/326 (52%)
HIV status				
	Adults aged >16 years with HIV	41/85 (48%)	48/71 (68%)	89/156 (57%)
	Children aged <16 years with HIV	5/170 (3%)	3/170 (2%)	8/170 (5%)
	Children aged <18 months exposed to HIV	14/107 (13%)	1/107 (1%)	15/107 (14%)
Current antiretroviral therapy*		35/46 (76%)	42/51 (82%)	77/97 (79%)
Current co-trimoxazole preventive therapy		49/60 (82%)	40/52 (77%)	89/112 (79%)
CD4 (cells per μL; median [IQR])		127 (22–359)	165 (38–293)	146 (41–340)

Data are median (IQR) or n/N (%).

* Denominators are the total number of adults and children living with HIV in each group.

**Table 2:** Bacteria isolated from participants with bloodstream infections and their resistance status to third-generation cephalosporins

	**Resistant to third-generation cephalosporins**	**Sensitive to third-generation cephalosporins**	**Overall**
*Escherichia coli*	76/220 (35%)	80/106 (75%)	156/326 (48%)
*Klebsiella pneumoniae*	111/220 (50%)	19/106 (18%)	130/326 (40%)
Other Enterobacterales	33/220 (15%)	7/106 (7%)	40/326 (12%)

Data are n/N (%). Data for other Enterobacterales are shown in appendix 1 (p8).

Participants were mainly young, with a median age of 13 years (IQR 1–42). 98 (30%) were young infants (<3 months) and, of these, 75 were neonates (<28 days). 169 (52%) of the 326 participants were male and 157 (48%) were female. 89 (57%) of 156 adult participants and 8 (5%) of the 170 children younger than 16 years were living with HIV (table 1). HIV prevalence was lower in adult participants with bloodstream infections that were resistant to third-generation cephalosporins (48%) than in those with bloodstream infections that were sensitive to third-generation cephalosporins (67%; table 1). Of 107 children younger than 18 months, 15 (13%) were HIV-exposed (ie, the mother was infected with HIV but the HIV antibody status of the child was unknown), 14 (93%) of whom had bloodstream infections that were resistant to third-generation cephalosporins. Coverage with antiretroviral therapy (77 [79%] of 97 patients) and co-trimoxazole preventive therapy (89 [79%] of 112 patients) was high; however, CD4 counts in adult participants were low (median 146 cells per μL [IQR 41–340]; table 1).

135 (41%) of the 326 participants died overall. The case fatality proportion was 45% (99 of 220) in patients with infections that were resistant to third-generation cephalosporins, and 34% (36 of 106) in patients with infections that were sensitive to third-generation cephalosporins. Resistance to third-generation cephalosporins was associated with an increased probability of death in hospital in model 1 (HR 1·44, 95% CI 1·02–2·04; table 3). Adjusting for effective antibiotic therapy (using model 2) reduced this effect (HR 1·23, 0·74–1·70; table 3).

**Table 3:** Multivariable associations with in-hospital mortality and hospital discharge from Cox proportional hazards models

	**Model 1**	**Model 2**
In-hospital mortality	1·44 (1·02–2·04)	1·23 (0·74–1·70)
Hospital discharge	0·31 (0·22–0·45)	0·31 (0·22–0·45)

Data are hazard ratio (95% CI). In model 1, explanatory variables were age and health-care exposure (previous surgery and previous hospitalisation); in model 2, explanatory variables were the same as in model 1 but with the addition of effective antibiotic use.

**Figure 1:**
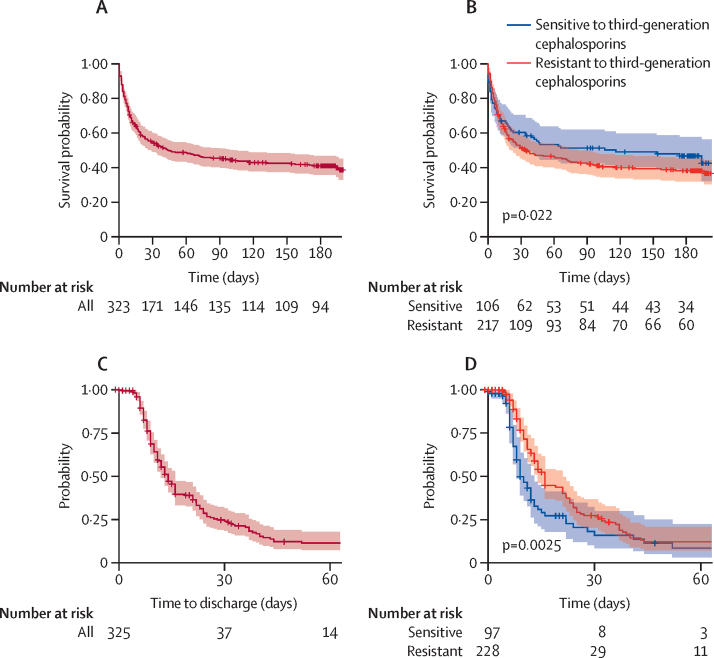
Kaplan-Meier curves for survival and time to discharge Kaplan-Meier survival curves of all participants (A) and participants stratified by third-generation cephalosporin resistance status (B). Crosses indicate censoring. The p value is derived from a log-rank test comparing the survival of participants with infections that were resistant and those with infections that were sensitive to third-generation cephalosporins. Outcome data were missing for three participants. Kaplan-Meier curves showing time to discharge for all participants (C) and participants stratified by third-generation cephalosporin resistance status (D). Crosses indicate censoring. The p value is derived from a log-rank test comparing length of hospital stay in participants with infections that were resistant and those with infections that were sensitive to third-generation cephalosporins. The date of discharge was missing for one participant.

The Kaplan-Meier estimation of survival function for all participants is shown in figure 1. Survival was improved in patients with infections that were sensitive to third-generation cephalosporins (median 116 days) compared with patients with infections that were resistant to third-generation cephalosporins (35 days). A rapid decline in survival was seen in the first 2 weeks after hospital admission, which then slowed to the end of the 180-day study period. Stratifying by third-generation cephalosporin resistance status showed that this early mortality was similar in patients with infections that were resistant to third-generation cephalosporins and in patients with infections that were sensitive to third-generation cephalosporins. Subsequent divergence of the curves suggests a higher probability of survival in patients with bloodstream infections that were resistant to third-generation cephalosporins after these early deaths, but there was no overall difference in survival on log-rank testing (p=0·22).

The median length of hospital stay was 7 days (IQR 2–12) for participants with bloodstream infections that were sensitive to third-generation cephalosporins and 8·5 days (2–36) for participants with bloodstream infections that were resistant to third-generation cephalosporins. With death considered as a competing risk, resistance to third-generation cephalosporins was associated with decreased probability of hospital discharge in model 1 and in model 2 (HR 0·31, 0·22–0·45 for both models; table 3). The Kaplan-Meier curves of time to discharge (measured from day of admission) are shown in figure 1. Early separation of the curves stratified by third-generation cephalosporin resistance status suggests that participants with infections that were resistant to third-generation cephalosporins required longer hospital stays, which was significant on log-rank testing (p<0·0025).

Recent (within the past 3 months) health-care exposure and hospital-onset bloodstream infections were more frequent in patients with bloodstream infections that were resistant to third-generation cephalosporins than in patients with infections that were sensitive to third-generation cephalosporins (table 4) and, excluding neonatal sepsis, 161 (59%) of 274 bloodstream infections were classified as health-care-associated overall. Epidemiological attribution by organism is shown in appendix 1 (p 8). Previous hospitalisation, recent surgery, and recent catheterisation were all more frequent in patients with bloodstream infections that were resistant to third-generation cephalosporins than in patients with infections that were sensitive to third-generation cephalosporins (table 4). Previous antimicrobial exposure was common in this cohort (103 [32%] of 326 patients), with no difference found between infections that were resistant or sensitive to third-generation cephalosporins.

**Table 4:** Bloodstream infections across the cohort by location of onset and health-care exposure

	**Resistant to third-generation cephalosporins**	**Sensitive to third-generation cephalosporins**	**Total**
Hospital-onset	106/274 (39%)	22/274 (8%)	128/274 (47%)
Community-onset, health-care-associated	23/274 (8%)	10/274 (4%)	33/274 (12%)
Community-onset, non-health-care-associated	50/274 (18%)	63/274 (23%)	113/274 (41%)
Early-onset neonatal sepsis	39/52 (75%)	10/52 (19%)	49/52 (94%)
Late-onset neonatal sepsis	1/52 (2%)	2/52 (4%)	3/52 (6%)
Previous hospitalisation*	58/220 (26%)	16/106 (15%)	74/326 (23%)
Previous surgery*	58/220 (26%)	8/106 (8%)	66/326 (20%)
Previous urinary catheterisation*	80/220 (36%)	23/106 (22%)	103/326 (32%)
Previous antibiotics*	65/220 (30%)	38/106 (36%)	103/326 (32%)

* Previous is defined as in the 3-month period before the onset of bloodstream infection.

Phenotypic antimicrobial susceptibility patterns were established for all isolates (appendix 1 p 9). All isolates were sensitive to meropenem. Ciprofloxacin resistance was detected in 155 (69%) of 226 isolates that were resistant to third-generation cephalosporins and in 18 (17%) of 106 isolates that were sensitive to third-generation cephalosporins. Chloramphenicol resistance was detected in 108 (48%) of 226 isolates that were resistant to third-generation cephalosporins and in 22 (20%) of 106 isolates that were sensitive to third-generation cephalosporins. Gentamicin resistance was high (187 [83%] of 226) in isolates that were resistant to third-generation cephalosporins and was lower (15 [14%] of 106) in isolates that were sensitive to third-generation cephalosporins, whereas amikacin resistance was low in all isolates, and was detected in 17 (8%) of 226 isolates that were resistant to third-generation cephalosporins and in only 2 (2%) of 106 isolates that were sensitive to third-generation cephalosporins.

The empirical antibiotic regimen contained an antibiotic that was active against the cultured bloodstream isolate (termed an effective antibiotic) in 87 (82%) of 106 bloodstream infections that were sensitive to third-generation cephalosporins but in only 25 (11%) of 220 bloodstream infections that were resistant to third-generation cephalosporins (appendix 1 p 10). The median time to effective antibiotic administration was 5 days (IQR 0–7) for patients with bloodstream infections that were resistant to third-generation cephalosporins. For all patients with infections that were sensitive to third-generation cephalosporins, an effective antibiotic was received on the day of admission. The most frequent antibiotic prescribed to patients with infections that were resistant to third-generation cephalosporins was ceftriaxone, which was prescribed to 164 (75%) of 220 patients. Carbapenems were used in only 34 (15%) of 220 bloodstream infections.

**Figure 2:**
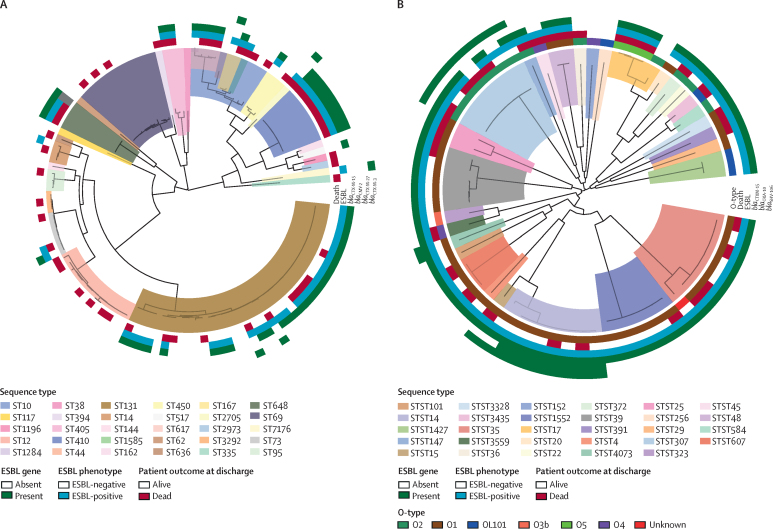
Midpoint-rooted maximum likelihood core-gene phylogenetic trees (A) Phylogenetic tree for *Escherichia coli.* Tree clades are coloured by sequence type; rings around the tree, from the inside out, show patient outcomes at discharge, extended-spectrum β-lactamase (ESBL) phenotype, and presence of ESBL genes. (B) Phylogenetic tree for *Klebsiella pneumoniae.* Tree clades are coloured by sequence type; rings around the tree, from the inside out, show O-antigen types (O-type), patient outcome at discharge, ESBL phenotype, and presence of ESBL genes.

119 *E coli* and 66 *K pneumoniae* genomes passed quality control for bioinformatic analysis. This analysis revealed a high diversity of both *E coli* and *K pneumoniae* genomes (figure 2). Among *E coli*, 32 sequence types (STs) were identified, the most common of which was ST131 (44%), followed by ST69 (12%) and ST410 (8%). Among 66 *K pneumoniae* genomes there were 29 sequence types, including ST14 (12%) and ST35 (12%). The most common gene encoding an extended-spectrum β-lactamase was *bla*_CTX-M-15_, both in *E coli* (present in 51% of genomes) and *K pneumoniae* (present in 89% of genomes). Other genes encoding extended-spectrum β-lactamases identified in the *E coli* collection include *bla*_CTX-M-27_ (3%) and *bla*_CTX-M-3_ (2%). For the *K pneumoniae* collection, other genes encoding extended-spectrum β-lactamases included *bla*_SHV-106_ (24%) and *bla*_OXA-10_ (12%). Complete information on sequence type and antimicrobial resistance genes are presented in appendices 2 and 3.

We reconstructed a core-gene phylogeny and linked it to data on patient outcome (dead or alive), sequence type, and presence or absence of genes encoding extended-spectrum β-lactamases. The phylogenetic clustering of isolates was consistent with sequence types for both *E coli* and *K pneumoniae*, although for some large sequence types—such as *E coli* ST131—isolates branched to form smaller subclades. The within-clade relative frequency of death was higher in some *E coli* clades than in others, and all patients from whom *E coli* ST410 (eight patients), ST617 (three patients), and ST648 (three patients) were isolated died. All isolates from these three sequence types had the *bla*_CTX-M-15_ gene, but additionally—and unique to the isolates in the clade—ST410 genomes also carried the cephalosporinase *bla*_CMY-2_ gene (figure 2). No such pattern was observed for *K pneumoniae*.

## Discussion

We show that patients with Enterobacterales bloodstream infections in Blantyre, Malawi, have poor clinical outcomes. However, patients with bloodstream infections that were resistant to third-generation cephalosporins had increased risk of death and longer hospital stays than patients with bloodstream infections that were sensitive to third-generation cephalosporins. To our knowledge, our study is the first description of the clinical burden in Malawi of bloodstream infections that are resistant to third-generation cephalosporins, and one of few such prospective studies from sub-Saharan Africa.^2^ As such, our data make a key contribution to our understanding of the burden of antimicrobial resistance in sub-Saharan Africa, and provide a platform for future antimicrobial resistance research in the region. The observed mortality in our study was extremely high when compared with that in high-income settings.^14^ Early recognition of sepsis, rapid diagnostics, and optimised antimicrobial strategies will be required to improve outcomes for patients with bloodstream infections in Malawi, in addition to strategies that address the control of antimicrobial resistance.^5^

Ceftriaxone is one of the most frequently used antibiotics for hospitalised patients in Malawi; its broad spectrum of activity, affordability to the health system, and once-daily dosing regimen make it a practical choice, especially where nursing capacity is low. However, ceftriaxone can no longer be the antibiotic of first and last resort in Malawi.^4^, ^5^ By linking quality-assured microbiological data to a prospective cohort, we highlight unacceptably poor patient outcomes. Our study therefore provides impetus for the allocation of resources towards mitigation strategies for both sepsis and antimicrobial resistance, including improved laboratory capacity, antimicrobial stewardship, and infection prevention and control.

The explanation for adverse outcomes for patients with bloodstream infections that are resistant to third-generation cephalosporins is likely to be multifactorial and could include both host and pathogen factors.^15^, ^16^ However, the finding that adjustment for effective antibiotic therapy reduces the association between resistance to third-generation cephalosporins and mortality supports the concept that poor outcomes in drug-resistant infections are in part mediated by a lack of appropriate antibiotic treatments. This finding has been documented elsewhere^17^ and, although perhaps unsurprising, is crucial to recognise and document in the context of Malawi, where third-generation cephalosporins are frequently the antibiotics of first and last resort, and where improving access to WHO Watch and Reserve antibiotics—such as carbapenems and aminoglycosides—could save lives.

The prevalence of HIV among adults in the cohort was high, and similar to that in other recent cohorts of patients with infection at QECH.^4^ Perhaps unexpectedly, HIV prevalence was lower in adult participants with bloodstream infections that were resistant to third-generation cephalosporins than in those with infections that were sensitive to third-generation cephalosporins; however, data describing the effect of HIV on resistance to third-generation cephalosporins remains scant.^18^ In children younger than 18 months, resistance to third-generation cephalosporins occurred more frequently in the HIV-exposed group than in the HIV-unexposed group, although small numbers make this finding difficult to interpret. In Malawi, around 5–8% of these HIV-exposed infants will test positive for HIV by the age of 24 months,^19^ but there is some suggestion that even children who are HIV-exposed but remain uninfected are immune compromised and have higher morbidity from infections.^20^ Perhaps young age (especially <1 month) and exposure to HIV have a combined immunomodulatory effect that is greater than that of exposure to HIV alone.

Before our study, it was assumed that the bloodstream infection surveillance data from Blantyre predominantly represented community-acquired infections;^6^ however, detailed characterisation of each bloodstream infection in our study showed that more than 60% of patients had recent exposure to health care. We did not power our study to determine risk factors for bloodstream infections that are resistant to third-generation cephalosporins but, as in other settings, we find that health-care exposures are potentially important determinants of drug-resistant infection.^21^, ^22^ Bloodstream infections that were resistant to third-generation cephalosporins were more likely to be hospital-acquired or health-care-associated than those that were sensitive to third-generation cephalosporins, and health-care factors such as previous use of urinary catheters, recent surgery, and recent hospitalisation were all more common in patients with bloodstream infections that were resistant to third-generation cephalosporins. Improved infection prevention and control programmes in health-care settings will therefore play a crucial part in antimicrobial resistance preventive strategies.

The diversity of sequence types represented in our collection are broadly representative of previously described *E coli* and *K pneumoniae* isolates associated with bloodstream infections and third-generation cephalosporin resistance both in Malawi and worldwide.^23^, ^24^ Clinical metadata on patient outcomes from global collections of Enterobacterales are often unavailable, especially in genomic studies. Our analysis of pathogen genomic data alongside patient outcomes reveals the circulation of sequence types such as *E coli* ST410, which in other settings was found to always produce extended-spectrum β-lactamases and contains a highly carbapenem-resistant clone.^25^, ^26^ Although our study was underpowered by the numbers within each sequence type to quantify the risk of mortality associated with these genotypes, the high prevalence of deaths among patients infected by these sequence types warrants further investigation.

Our study had some limitations. First, recruitment was limited to one hospital owing to the availability of blood-culture facilities. A deeper understanding of the burden of antimicrobial resistance will be developed by including other pathogens and clinical syndromes; larger cohorts to permit age stratification; and routine, clinically orientated surveillance that links laboratory data to patient outcomes.^27^ Second, the presence of co-infection with other pathogens in advanced HIV (eg, disseminated *Mycobacterium tuberculosis* infection) could worsen outcomes in patients with bloodstream Enterobacterales infections, and these data should ideally be conditioned upon in outcome models. Finally, this study contained insufficient data to include a sepsis severity variable as an adjustment factor in the models, as several of the domains of sepsis scores require parameters that are not available at QECH or require ventilation and inotropic support, which is not routinely assessed.^28^

To our knowledge, we describe the first morbidity and mortality burden estimates in Malawi of bloodstream infections that are resistant to third-generation cephalosporins, generated using standardised methodology in line with WHO Global Antimicrobial Resistance and Use Surveillance System recommendations.^29^ We find that bloodstream infections with Enterobacterales are associated with high mortality overall, but that resistance to third-generation cephalosporins exacerbates the risk of death and leads to longer hospital stays. Furthermore, we identify that health-care exposure might have an important role in the risk of developing drug-resistant infections. Our data advance the emerging literature on the burden of antimicrobial resistance in Africa, and will contribute to making the practice of estimating outcomes in low-income and middle-income countries by extrapolation from high-income settings redundant.

## Data sharing

Genome sequence data included in this study are available at the European Nucleotide Archive (https://www.ebi.ac.uk/ena) under accession numbers listed in appendices 2 and 3. The study protocol is published in the reviewed literature.^11^

## Declaration of interests

NAF received a Wellcome Asia and Africa Programme Grant to the Malawi Liverpool Wellcome Research Programme and a Medical Research Council programme grant. All other authors declare no competing interests.
