# Supplementary material for: Effect of resistance to third-generation cephalosporins on morbidity and mortality from bloodstream infections in Blantyre, Malawi: a prospective cohort study
Source: Lancet Microbe. 2022 Dec;3(12):e922–30. doi: 10.1016/S2666-5247(22)00282-8 (PMC9712123; doi:10.1016/S2666-5247(22)00282-8)
Supplement: Supplementary appendix [file mmc1.pdf]

THE LANCET
Microbe

Supplementary appendix 1

This appendix formed part of the original submission and has been peer reviewed. We post it as supplied by the authors.

Supplement to: Lester R, Musicha P, Kawaza K, et al. Effect of resistance to third-generation cephalosporins on morbidity and mortality from bloodstream infections in Blantyre, Malawi: a prospective cohort study. *Lancet Microbe* 2022; published online Nov 3. https://doi.org/10.1016/S2666-5247(22)00282-8.

Supplementary Material

Supplementary Table 1. Definitions of epidemiological classifications.[1,2]

Category	Definition
Community onset, healthcare-associated	BSI which develops in the community, in a patient who has received healthcare in either the community or hospital in the previous 28 days
Community onset, non-healthcare-associated	BSI which develops in the community, in a patient who was not known to have attended healthcare facilities in either the community or hospital in the previous 28 days
Hospital onset	BSI which develops in a patient 48 hours or more after admission to hospital (either QECH or a hospital from which the patient was directly transferred to QECH, without spending a night at home)
Early-onset neonatal sepsis	Bloodstream infection occurring in young infants in the first week of life
Late-onset neonatal sepsis	Bloodstream infection occurring in young infants from one week to 3 months of life

Supplementary Methods

Approach to variable selection using directed acyclic graph (DAG)

The aim of our analysis was to estimate the effect of 3GC-R on mortality. The purpose of the DAG was to identify a minimum adjustment set to minimise bias when estimating causal effect. Based on the DAG diagram, the minimum adjustment set for the total effect of 3GC-R on death (closure of only biasing paths, shown as dotted edges) requires adjustment for age, HIV and healthcare exposures. To estimate the direct effect of 3GC-R on death, all other biasing and causal paths must be closed, leaving only the edge from 3GC-R to death (shown in red). To estimate the total effect of 3GC-R on death, the minimum adjustment set is therefore age, HIV and healthcare exposures. To estimate the direct effect of 3GC-R on death would additionally require adjustment for sepsis severity, other pathogens and treatment with an effective antibiotic.

Multivariable models which adjusted for relevant confounders were then built, to investigate the causal relationship between 3GC-R and mortality. I used a causal inference framework to identify potential confounders in these models. Briefly, a causal diagram, known as a directed acyclic graph (DAG), representing the relationships between 3GC-R and death was drawn using a causal inference framework.[3,4] The dagitty package in R[4] was used to automate the framework, in order to determine a minimum adjustment set of conditioning variables. The identified variables were used in subsequent Cox-proportional hazard models.

Two models were fitted. The first model (base model) aimed to estimate the total effect of 3GC-R on mortality/length of stay and conditioned upon age, HIV and healthcare exposures. Healthcare exposures were selected if they were associated with 3GC-R on univariate analysis and if complete data were available for the variable. These variables were therefore prior operations and prior hospitalisation.

The second model aimed to condition upon other pathogens, sepsis severity and treatment with effective antibiotics, in order to estimate the direct effect of 3GC-R on mortality/length of stay. Effective antibiotic usage data was included in the models as a binary covariate (use of effective antibiotic or not) as only 7.7% of participants received no antibiotic, meaning there would be insufficient heterogeneity to include this in the covariate. There were too many missing data to include a sensible "sepsis severity" covariate in the models. In terms of "other pathogens", MTB is the most likely to be present in this cohort, but accurate TB diagnostics were not available at QECH at the time the study was recruiting and these data are again missing. Because of these limitations, the second model conditioned upon age, prior operations, prior hospitalisation and effective antibiotic use only.

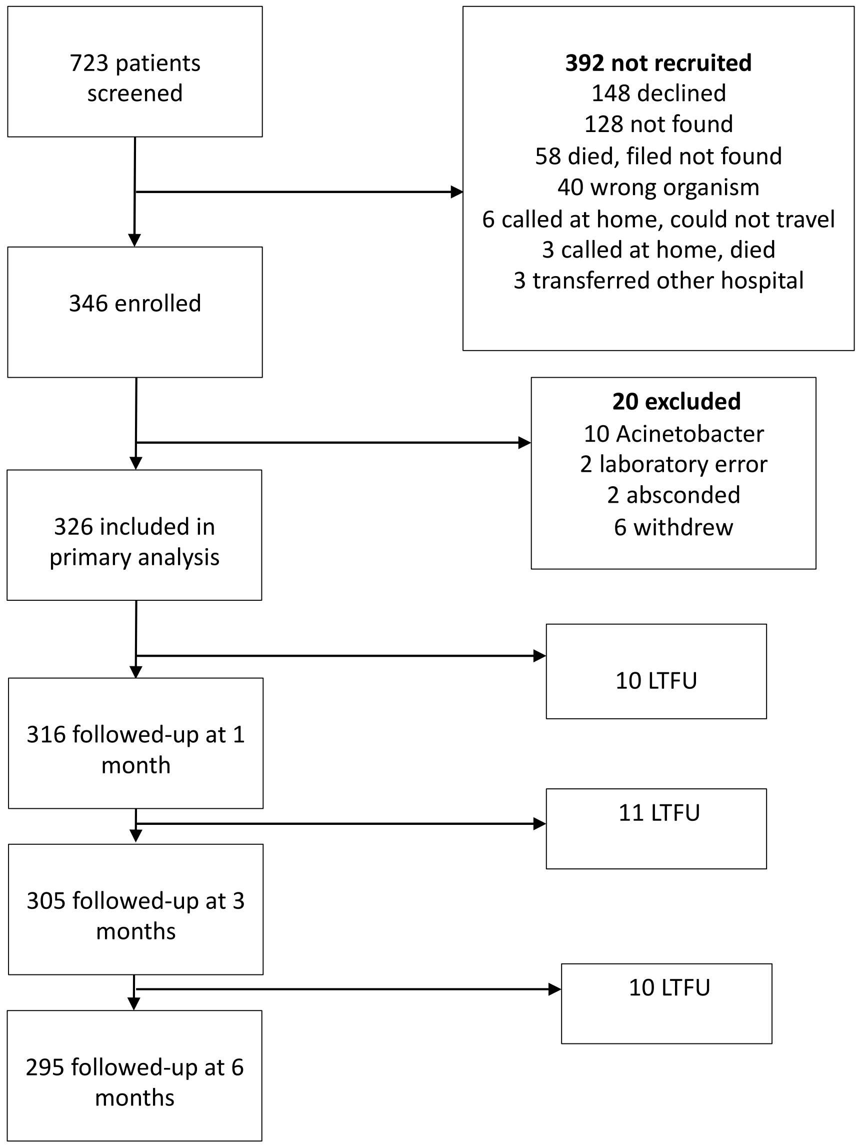

Supplementary Figure 1. Study population at recruitment and follow-up. LTFU = lost to follow-up

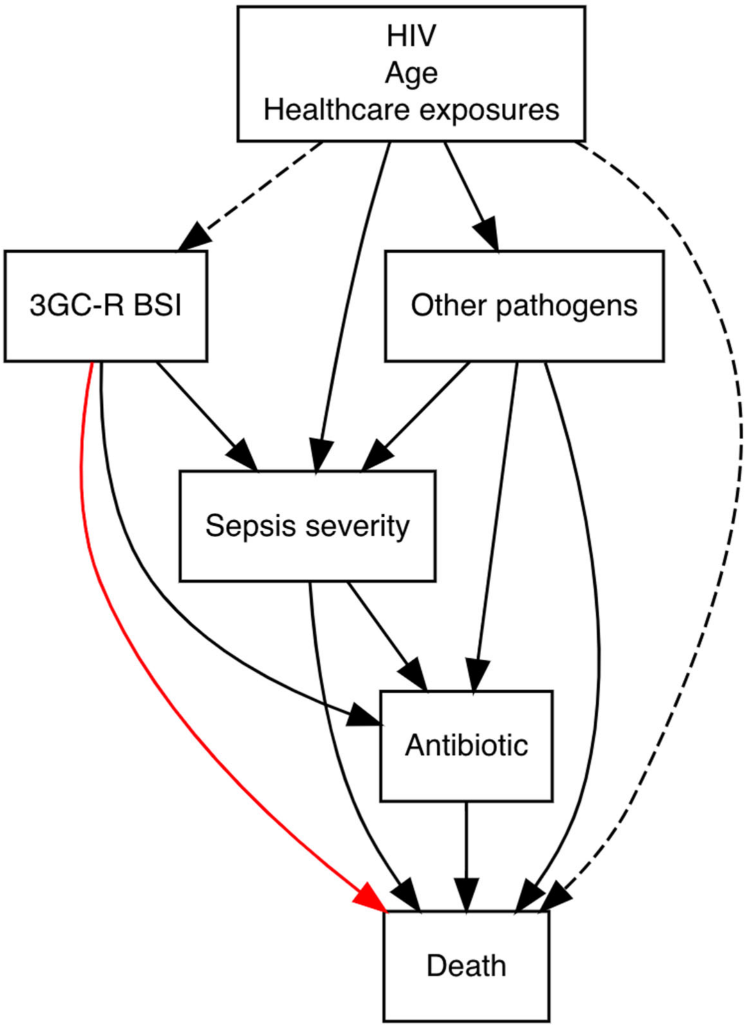

Supplementary Figure 2. Hypothesised causal diagram for causal effect of 3GC-R BSI on death. Causal paths are shown as arrows. Host-factors such as HIV, age and prior healthcare exposure are exogenous factors affecting both the exposure (3GC-R) and outcome (Death) and are therefore potential confounded associations (biasing paths) shown as dotted arrows (or edges). Red arrow indicates direct causal effect of 3GC-R on death. Death from 3GC-R is mediated in part by sepsis which may vary in severity or be absent (sepsis severity). Sepsis severity may influence the antibiotic given which in turn may be active against the infection, not active or not given at all. Other infectious pathogens may influence sepsis severity, treatment choice and outcome. To estimate the total effect of 3GC-R on death, only biasing pathways must be closed. To estimate the direct effect of 3GC-R on death (red arrow), all other pathways must be closed.

Whole-genome sequencing and quality control

Genomic DNA was extracted from 313 unique Blood culture isolates at the Malawi-Liverpool-Wellcome Research Programme (MLW) using Qiagen DNA mini kit (Qiagen, Germany) as per the manufacturer's instructions. Genomes were sequenced at the Wellcome Sanger Institute using the Illumina X10 platform (Illumina, Inc., San Diego, California) to generate paired end reads of 150bp length. Quality of raw reads was assessed using FastQC (https://www.bioinformatics.babraham.ac.uk/projects/fastqc/) and MultiQC.[5] We checked for contamination using Kraken v0.10.6 and excluded samples with < 70% of reads of either E. coli or K. pneumoniae.

***De novo* assembly and sequence annotation**

We used Spades (v3.14.0)[6] to assemble raw reads into contiguous sequences (contigs) and filtered out all contigs of length <300bp. Assemblies with total length < 4BM or > 6MB were excluded from further analysis. Assemblies were annotated using PROKKA.[7] Raw sequence data were deposited in the European Nucleotide Archive (ENA) and ENA accession numbers are included in Table S1.

Phylogeny reconstruction

We used the Roary[8] to construct a core genome of the annotated genome assemblies separate for each collection of *E. coli* and *K. pneumoniae*. For each of E. coli and K. pneumoniae, core gene alignments were then generated through concatenation of the alignments of orthologous core genes. We extracted single nucleotide polymorphic (SNP) sites from the core genome alignment using snp-sites[9] to generate a core-snp alignment for each of the *E. coli* and *K. pneumoniae* genome collections. Maximum likelihood (ML) phylogenetic trees were constructed from either of the SNP alignments using IQ-TREE with GTR+I+G model.[10] Reliability of the inferred branches and branch partitions in the phylogenetic trees were assessed using 1000 bootstrap replicates. Phylogenetic trees were visualised and annotated using ggtree R package.

***In silico* multilocus sequence typing and identification of AMR genes**

E. coli MLST was performed using mlst tool (v2.16.2) (https://github.com/tseemann/mlst). For *K. pneumoniae* isolates MLST was performed using Kleborate 2.1[11] with the kaptive option turned on for identification of k-

and o- antigen We screened for the presence of acquired AMR genes using ABRIcate (https://github.com/tseemann/abricate) against the NCBI AMR database. In all cases, we used minimum thresholds of 95% blastp identity and 90% coverage.

Supplementary Results

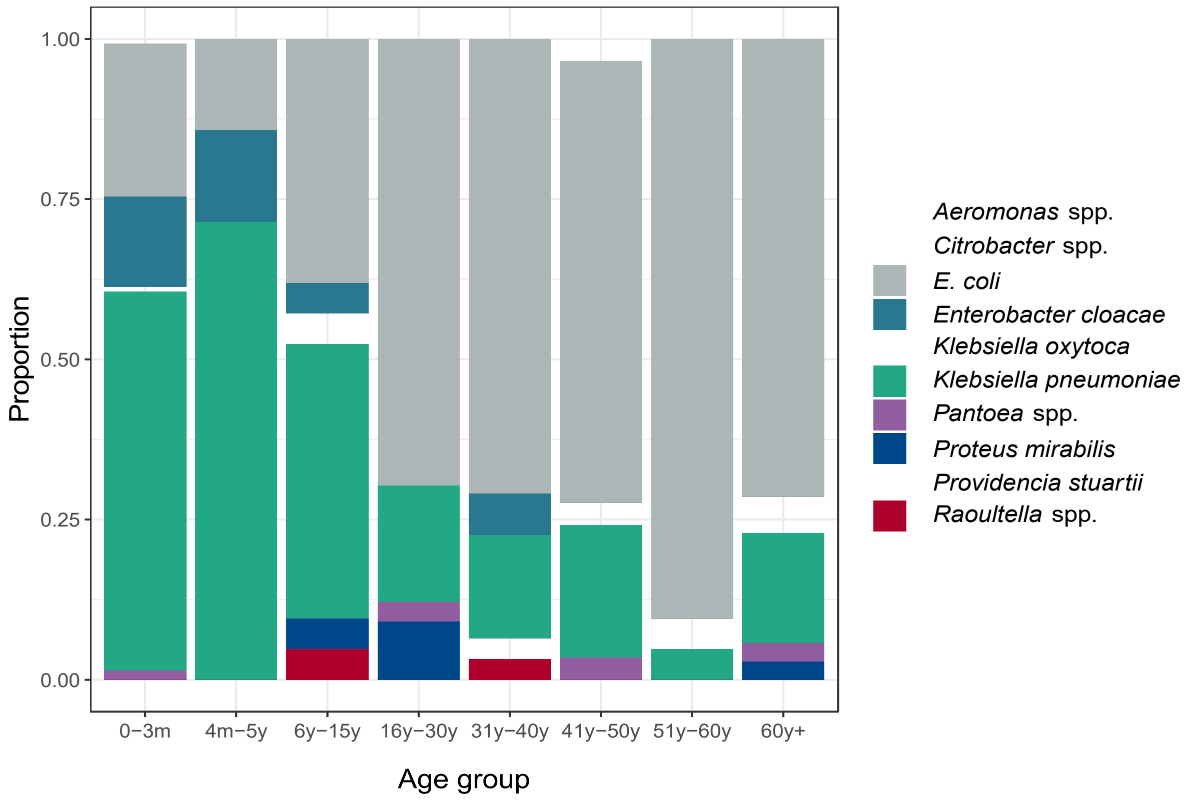

Supplementary Figure 3. Bloodstream infection stratified by age group.

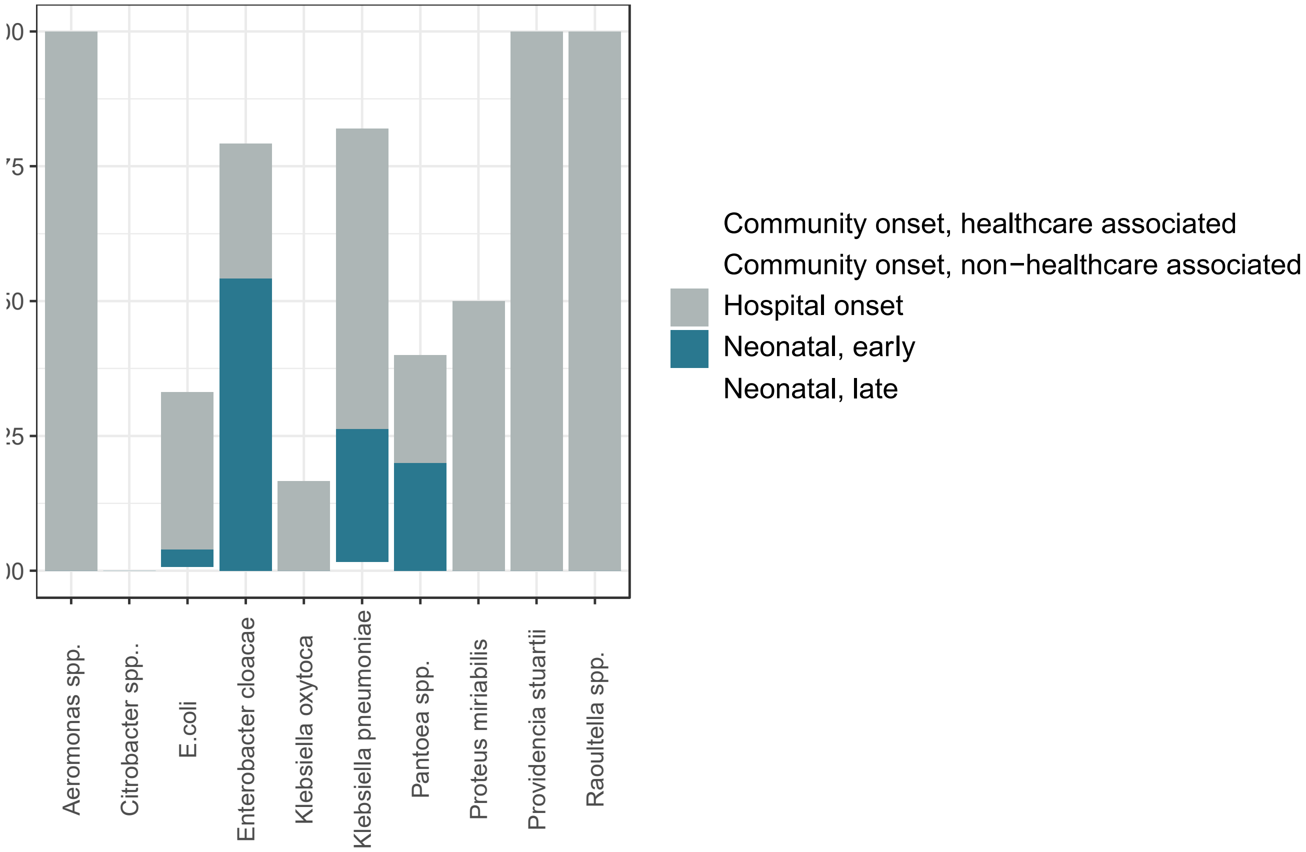

Supplementary Figure 4. BSI organism stratified by epidemiological classification.

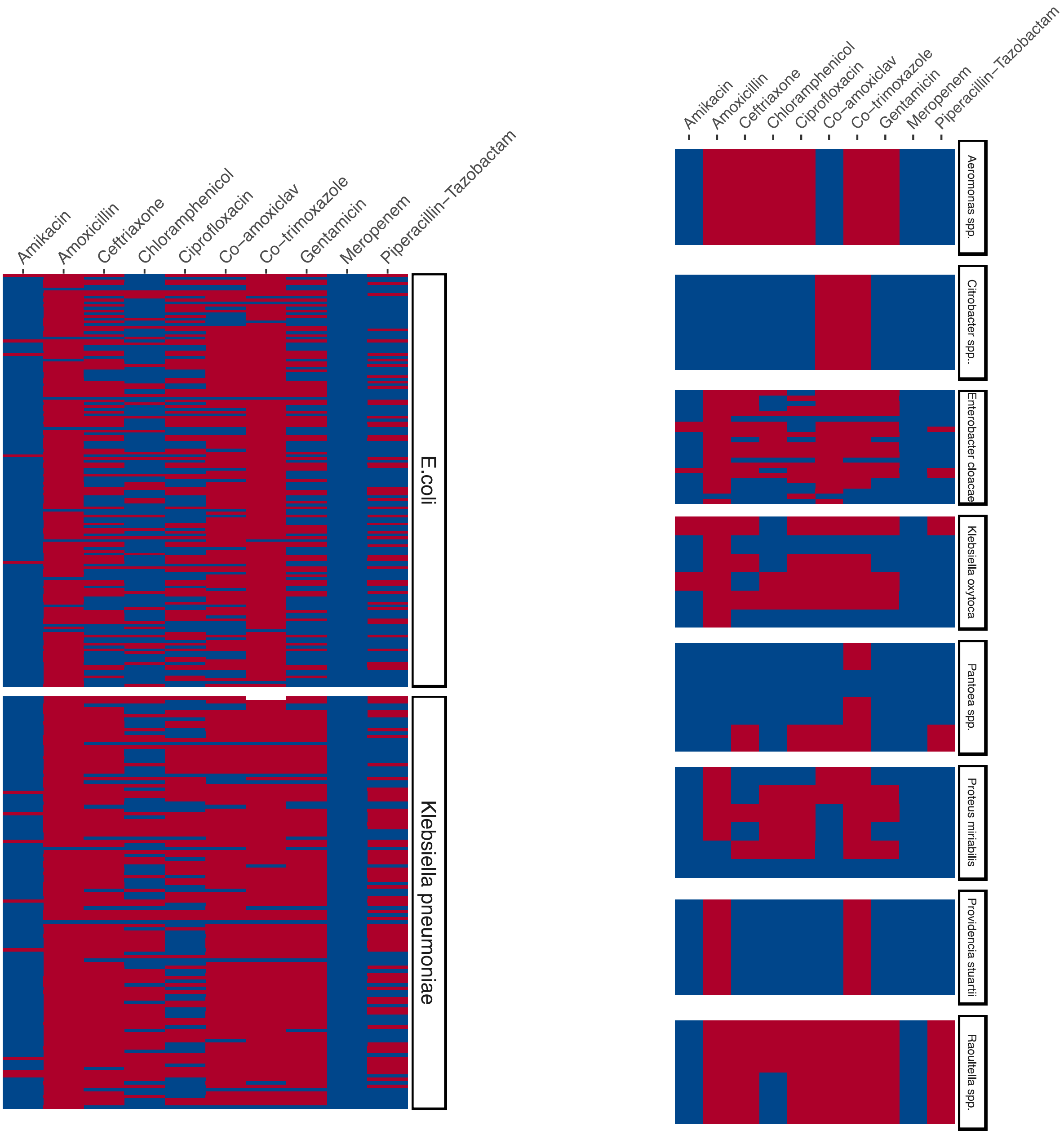

Supplementary Figure 5. Phenotypic antimicrobial resistance profiles for all tested isolates using EUCAST breakpoints. Each row is one isolate and each column is the tested antimicrobial, Red = resistant, blue = sensitive.

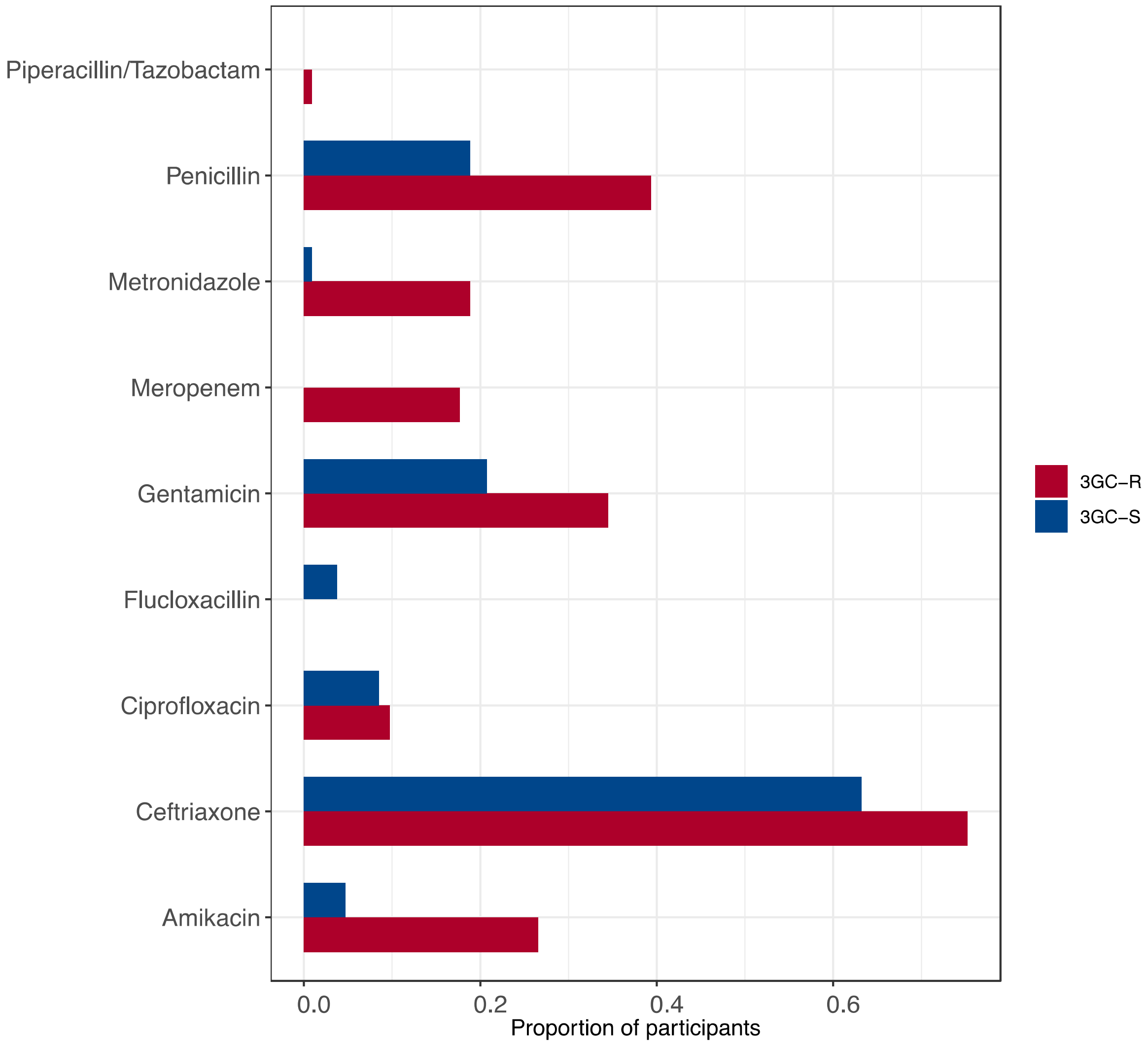

Supplementary Figure 6. Antibiotics used for treatment of BSI in the cohort.

Supplementary Table 2: Results of sensitivity analysis

Multivariable associations with in-hospital death and hospital discharge from Cox proportional hazards model

	Base Model (Model 1)	Model 2
	HR	HR
In-hospital mortality	1.46 (1.03-2.06)	1.24 (0.74-1.71)
Hospital discharge	0.30 (0.22- 0.46)	0.30 (0.23-0.45)

Supplementary References

1. Civitarese AM, Ruggieri E, Walz JM, et al. A 10-Year Review of Total Hospital-Onset ICU Bloodstream Infections at an Academic Medical Center. *Chest* 2017; **151**(5): 1011-7.
2. Fuchs A, Bielicki J, Mathur S, Sharland M, Van Den Anker JN. Reviewing the WHO guidelines for antibiotic use for sepsis in neonates and children. *Paediatrics and international child health* 2018; **38**(sup1): S3-S15.
3. Joseph W. Hogan, 2019. "Causal inference in statistics: A primer. Judea Pearl, Maria Glymour, and Nicholas Jewell, John Wiley & Sons, Ltd., Chichester, UK," Biometrics, The International Biometric Society, vol. 75(2), pages 708-709, June.
4. Johannes Textor and Benito van der Zander (2016). dagitty: Graphical Analysis of Structural
Causal Models. R package version 0.2-2. https://CRAN.R-project.org/package=dagitty.
5. Ewels P, Magnusson M, Lundin S, Kaller M. MultiQC: summarize analysis results for multiple tools and samples in a single report. *Bioinformatics* 2016; **32**(19): 3047-8.
6. Bankevich A, Nurk S, Antipov D, et al. SPAdes: a new genome assembly algorithm and its applications to single-cell sequencing. *J Comput Biol* 2012; **19**(5): 455-77.
7. Seemann T. Prokka: rapid prokaryotic genome annotation. *Bioinformatics* 2014; **30**(14): 2068-9.
8. Page AJ, Cummins CA, Hunt M, et al. Roary: rapid large-scale prokaryote pan genome analysis. *Bioinformatics* 2015; **31**(22): 3691-3.
9. Page AJ, Taylor B, Delaney AJ, et al. SNP-sites: rapid efficient extraction of SNPs from multi-FASTA alignments. *Microb Genom* 2016; **2**(4): e000056.
10. Nguyen LT, Schmidt HA, von Haeseler A, Minh BQ. IQ-TREE: a fast and effective stochastic algorithm for estimating maximum-likelihood phylogenies. *Mol Biol Evol* 2015; **32**(1): 268-74.
11. Lam MMC, Wick RR, Watts SC, Cerdeira LT, Wyres KL, Holt KE. A genomic surveillance framework and genotyping tool for Klebsiella pneumoniae and its related species complex. *Nat Commun* 2021; **12**(1): 4188.
